# Retinal waves are likely to instruct the formation of eye-specific retinogeniculate projections

**DOI:** 10.1186/1749-8104-4-24

**Published:** 2009-07-06

**Authors:** Marla B Feller

**Affiliations:** 1 Department of Molecular and Cell Biology and Helen Wills Neuroscience Institute University of California, Berkeley, Berkeley, California 94720, USA

## Abstract

Prior to eye-opening and the development of visual responses, the retina exhibits highly correlated spontaneous firing pattens termed retinal waves. Disruption of the normal spontaneous firing pattern either genetically or pharmacologically prevents the eye-specific refinement of retinogeniculate afferents. Here I provide the evidence that retinal waves play an instructive role in this process. In addition, I argue that a full understanding requires an identification of the features of retinal activity that drive the refinement as well as an understanding of mechanisms that transform these signals into axonal rearrangements.

## Introduction

A fundamental feature of the developing neural circuits is their ability to change in the presence of altered sensory experience. This phenomenon has been studied particularly in the generation of cortical maps, where altering the pattern of sensory experience alters the spatial organization of sensory representations. The cellular mechanisms that underlie cortical plasticity fall into two categories: those that are based on physiological mechanisms, such as mechanisms by which altered firing patterns alter synaptic strength; and those that are based on morphological changes that are responsible for the physical rewiring of neural circuits. Physiological-based mechanisms that have been implicated in cortical plasticity include synaptic modifications such as long-term potentiation and long-term depression, which are often referred to as Hebbian-based learning rules. In addition, there are several non-Hebbian learning rules that have also been implicated, such as homeostatic plasticity and changes in intrinsic excitability of neurons (for extensive review of these mechanisms see [1] and references therein).

There is growing evidence that neural activity plays a role much earlier in development, prior to the maturation of the sensory epithelium. In several developing circuits, including the cochlea [2], spinal cord [3,4], hippocampus and cortex [5,6], there are transient features that cause these circuits to spontaneously generate correlated activity [7,8]. However, the cellular mechanisms that translate these spontaneous activity patterns into mature neural circuits are not well understood. Indeed, application of the physiological-based mechanisms identified during later cortical plasticity may not readily apply in circuits where synapses are first forming and, therefore, are relatively immature [9]. Hebbian-based learning rules are particularly popular for early development because they instruct which synapses stay and which ones go in that strong co-activation of pre- and postsynaptic cells causes synapse strengthening (long-term potentiation) while uncorrelated firing leads to synapse weakening (long-term depression). These contingencies have been expressed popularly by the phrases 'neurons that fire together wire together' and 'neurons not in synch lose their link' [10]. However, stringent tests of these ideas have been difficult for studies of spontaneous activity, for at least two reasons. First, the timescale over which the firing of neurons needs to be 'synchronized' is not well-defined. Second, manipulating patterns of activity without also affecting the total level of activity has proven to be challenging.

One system in which such tests have been attempted is the developing visual system. Prior to maturation of the light response, retinas exhibit a spontaneous firing pattern termed retinal waves. Retinal waves are a robust feature of the developing retina and have been observed in a wide variety of vertebrate species [11,12]. Retinal waves are composed of spontaneous bursts of action potentials that initiate in random locations and propagate across the developing inner and outer retina, eventually encompassing hundreds of cells. In mice, retinal waves are first detected a few days before birth and persist for approximately 2 weeks after birth, disappearing around the time of eye-opening. During the period of retinal waves, the retina itself is rapidly developing as different synaptic circuits wire up. These changes in retinal circuitry are reflected by changes in the mechanisms that mediate waves (for a review, see [2]). For the purposes of this debate, we will confine our discussions to the role of cholinergic waves since these occur between postnatal day 0 and postnatal day 10, which is the period of development when retinofugal maps are forming and when most experiments have been done.

Retinal waves provide a robust signal that drives activity in the dorsal lateral geniculate nucleus of the thalamus (dLGN) [13] and primary visual cortex [14]. Indeed, spontaneous retinal activity has been implicated in several aspects of visual system development, including the maturation of retinal ganglion cell (RGC) dendrites, the refinement of retinofugal projections into orderly maps and the establishment of ocular dominance columns and retinotopy in primary visual cortex (for reviews, see [15-17]).

The focus of the debate here is whether retinal waves play an 'instructive' role in the establishment of eye-specific layers in the dLGN. The term 'instructive' implies that the organizational features of the resulting circuit are determined by the spatial and temporal properties of the activity. This is in contrast to a 'permissive' role, a term that implies activity is required for basic neuronal function and growth but the pattern of activity is not critical. I prefer using the term 'inductive' over 'permissive', as suggested in a recent review [18], to include the possibility that patterned activity is critical to the formation of visual maps by regulating some cellular process, such as the expression of particular transcription factors that are the basis of map formation.

To illustrate the differences between instructive and permissive mechanisms, consider the formation of retinotopic maps in the superior colliculus. In the adult, neighboring RGCs project to neighboring cells in the superior colliculus, preserving the spatial organization of the retina. This connectivity pattern emerges during development from an initial pattern where RGCs project over significantly larger areas of the superior colliculus. An activity pattern that is instructive for the wiring of this map must contain information about the distances between two RGCs. During waves, the bursts of action potential of two nearby RGCs are more highly correlated than two distant RGCs; therefore, retinal waves could be instructive for retinotopic maps [19]. Retinal waves may also be inductive for retinotopic map formation in that they drive periodic increases in cAMP, which are required for growth cone repulsion after exposure to ephrin-A ligands [20]. In this scenario, the instructive for the retinotopic map is not contained in the activity pattern but rather in the concentration gradient of ephrins.

Here I review the evidence that retinal waves play an instructive role specifically in eye-specific segregation of retinal projections to the dLGN, which I will refer to as eye-specific layers. The strategy that has been used to test whether retinal waves play an instructive role is to alter the endogenous firing pattern during the period of development when maps are forming. I am restricting the discussion to manipulations that alter retinal firing patterns and not manipulations that may alter the read-out of these patterns, namely the retinofugal synapses (for review, see [17]). Three methods have been used to alter retinal waves *in vivo*: pharmacologically using intraocular injections of substances known to alter wave patterns *in vitro*; using immunotoxins that kill a specific classes of amacrine cells that are critical for generating retinal waves; and genetically, using knockout mouse lines that have altered spontaneous firing patterns. Below I summarize the findings using these three methods, the subsequent interpretations and the caveats for each experiment that limit our abilities to make strong conclusions.

## Some manipulations that alter retinal waves sometimes prevent the formation of eye-specific layers, while others do not

An effective pharmacological manipulation to alter spontaneous firing patterns during the first postnatal week is intraocular injections of the nicotinic acetylcholine receptor agonist epibatidine, which reliably prevents eye-specific segregation of retinogeniculate projections [21-25]. (Note, intraocular injections of epibatidine have been used to test other aspects of visual system development not covered here.) Based on whole cell physiology and imaging experiments in ferret [23] and mice [26], I and colleagues concluded that epibatidine blocks all spontaneous firing in the retina during the first postnatal week, when eye-specific layers are forming [23]. However, more recent studies using a multi-electrode array (MEA) to record simultaneously from tens to hundreds of neurons found that epibatidine blocked spontaneous spiking in roughly 50% of all RGCs and led to uncorrelated firing in the other 50% [27,28]. These results from MEA recordings point to limitations in the previous methods – whole cell current clamp recordings were done from RGCs with larger somas and, therefore, we were likely to be selectively recording from one of the types of RGCs whose membrane depolarizations were blocked by epibatidine. Fluorescence signals recorded during calcium imaging experiments were spatially and temporally filtered to monitor correlations induced by waves over large areas of the retina at the expense of monitoring single RGC activity. Hence, MEA recordings are more likely to reflect the true firing pattern of individual RGCs exposed to epibatidine *in vivo*.

Since intraocular injection of epibatidine prevents eye-specific layer formation, we can conclude that spontaneous retinal activity plays some role in map formation. However we cannot distinguish from these data alone whether retinal activity plays an inductive or instructive role. The observation that 50% of RGCs still fire robustly in the presence of epibatidine but with no correlated structure could be interpreted as proof that retinal waves are instructive for eye-specific map formation. However, the observation that epibatidine reduces firing in 50% of RGCs could be interpreted as proof that retinal waves are inductive or instructive for eye-specific map formation.

Immunotoxin treatments have several advantages over pharmacological treatments in that they are likely to be longer lasting and recordings from treated retinas can test their effectiveness. (Pharmacological treatments can wash out during the time of acute dissections and recordings.) Immunotoxins targeted to starburst amacrine cells, the retinal interneuron that is responsible for cholinergic retinal waves, were used by the Chapman and Chalupa labs to kill a large percentage of starburst amacrine cells [22]. By conducting simultaneous whole cell current clamp recordings from pairs of RGCs, they found that this treatment led to a significant reduction in nearest neighbor correlations. Interestingly, despite this disruption in the correlated firing, they found that treated animals had normal eye-specific layers in the dLGN. Based on these findings, the authors concluded that retinal waves are not instructive for eye-specific map formation but rather are inductive. However, calcium imaging in immunotoxin-treated ferret retina revealed that they exhibited propagating waves, indicating that there remained some correlation structure in the remaining firing pattern. Hence, another interpretation of these findings is that the specific aspect of activity disrupted by the immunotoxin manipulation was not instructive for eye-specific segregation, though the remaining correlated firings might be.

The third method for altering spontaneous firing patterns is to use knockout mice that have altered spontaneous firing patterns. The primary model my laboratory has used is a mouse that lacks the neuronal nicotinic acetylcholine receptor beta-2 subunit (β2-nAChR-KO). β2-nAChR-KO mice have significantly reduced eye-specific and retinotopic refinement, nearly identical to the results observed with epibatidine intraocular injections [25,29]. We reported that these mice have no correlated waves as assayed by calcium imaging [26] and MEA recording [30]. Rather, β2-nAChR-KO retinas have a smaller percentage of RGCs that spontaneously depolarize, and those RGCs that do depolarize have significantly reduced nearest neighbor correlations and lower firing rates during bursts. As with the epibatidine results, the lower firing rates and smaller percentage of active cells are consistent with either an inductive or instructive role for retinal activity in map formation while the uncorrelated firing is consistent with an instructive role.

The interpretation of the altered visual maps detected in β2-nAChR-KO mice have been called into question because recent reports indicate that, under some conditions, β2-nAChR-KO mice exhibit propagating activity [31]. This recent study confirms previous studies from my laboratory that demonstrated that, under certain pharmacological conditions, β2-nAChR-KO mice can support waves mediated by non-synaptic mechanisms [32]. Only experiments conducted *in vivo*, at normal body temperature, in a normal chemical environment and in the absence of anesthetics will determine whether the firing patterns described in the recent study or those described in several previous reports are more accurate.

## Identification of features of retinal waves that drive eye-specific segregation

Despite the pitfalls of the various manipulations described above, there are some observations that are undisputed. β2-nAChR-KO mice have altered eye-specific maps that are reproduced by intraocular injections of nAChR antagonists during the exact period of development that β2-nAChR-KO mice have abnormal firing patterns. Blockade of cholinergic waves in ferrets also leads to a permanent disruption in the formation of eye-specific layers similar to those observed in mice. Several other knockout mice that have altered signaling at retinogeniculate synapses also have disrupted eye-specific layers (reviewed in [17]). Therefore, the evidence is fairly compelling that disrupting the endogenous firing patterns disrupts the formation of eye-specific regions in the dLGN. Since these disruptions of pattern occur while a substantial level of activity remains, it is likely that activity plays an instructive rather than an inductive role.

If it is the pattern of retinal activity, and not activity in general, that drives eye-specific segregation, then the question becomes what specific aspects of the spatial correlations and temporal structure of retinal waves are instructive for map refinement. For example, even if it is determined that β2-nAChR-KO mice have waves *in vivo*, it does not mean that the waves observed in wild-type mice are not instructive for eye-specific segregation. Rather, it would provide additional clues as to what are the key features provided by waves to drive eye-specific segregation. This is perhaps most easily illustrated by considering retinotopic refinement. A feature of retinal waves that is instructive for retinotopic refinement is propagation speed. Waves in β2-nAChR-KO mice propagate at a much higher velocity and over longer distances and, therefore, cells that are located at long distances (even as far as 1 mm apart) are strongly correlated in the firing, while the nearest neighbor correlations in wild-type mice drop off with distance much more dramatically. Hence, the slower propagation speed of waves in wild-type mice may be instructive for formation of retinotopic maps.

What features of retinal waves are instructive for eye-specific segregation? We can start to answer this question by comparing the effects on eye-specific segregation of three manipulations that preserve waving activity but disrupt specific features of waves. First, intraocular injection of cAMP agonists in both eyes increases the frequency of retinal waves such that the inter-wave interval decreases from control retina values of 100 seconds to 75 seconds, but does not alter eye-specific segregation [33]. Second, knockout mice lacking the gap junction protein Cx36 exhibit significantly more asynchronous action potentials between waves than wild-type mice, but have normal eye-specific segregation [34]. Third, the no b-wave mouse, which has a spontaneous mutation in a protein in dendrites of bipolar cells, exhibits retinal waves that occur with an inter-wave interval of 15 seconds and fails to maintain segregation of eye-specific layers [35]. From these studies we hypothesize that the generation and maintenance of eye-specific layers may require intervals between waves that last longer than 15 seconds but can be less than 75 seconds, and that uncorrelated firing between waves does not inhibit formation of the map. How the β2-nAChR firing patterns contribute to this hypothesis is less clear. We reported that non-synaptic waves induced in β2-nAChR-KO mice have significantly different spatial correlations and bursting properties from wild-type retinas, and were not sufficient to rescue eye-specific refinement of retinogeniculate axons [36], consistent with the hypothesis that the endogenous pattern is critical for eye-specific refinement.

## The resolution lies in understanding the mechanisms that read-out retinal waves

Ultimately, to understand how waves contribute to eye-specific layer segregation will require an understanding of the mechanisms by which neural activity drives the rearrangement of axons that underlie map refinement. Though there is evidence that there is synaptic competition [37,38], a deeper understanding of the learning rules that drive this competition needs to be gained [9]. In addition, there is clearly some interaction between activity and ephrin-mediated repulsion [20,39], indicating that activity in individual RGCs, and not just competitive interactions, is also important for the establishment of maps. Thus, the question of whether waves are instructive or inductive may represent a false dichotomy.

It is time to move away from the dogmatic approach of the old 'nature versus nurture' debate. As more RGC cell type-specific factors are identified and our ability to make subtle activity manipulations grows, we will be able to formulate specific hypotheses regarding the role of patterned activity in specific aspects of visual system development.

## Abbreviations

β2-nAChR-KO: neuronal nicotinic acetylcholine receptor beta-2 subunit knockout; dLGN: dorsal lateral geniculate nucleus; MEA: multi-electrode array; nAChR: neuronal nicotinic acetylcholine receptor; RGC: retinal ganglion cell.

## Competing interests

The author declares that they have no competing interests.
